# The Alvarenga Prize

**Published:** 1892-08

**Authors:** 


### THE ALVARENGA PRIZE.

It is with much pride and gratification that the JOURNAL announces the award of the Alvarenga cash prize for 1892 to Dr. R. H. L. Bibb, of Saltillo, Mexico.

Dr. Bibb is a Texan, and for many years has been closely identified with the Texas profession. Residing in Austin formerly, he was extensively known,—his reputation as a surgeon being co-extensive with the State bounds. He was largely instrumental in the organization and building up of the State Medical Association, having served some years as its secretary,—and contributed many valuable papers to its published Transactions. He is perhaps better informed upon the history of this organization than any man living; in fact, he has written out a history of the Association, which should, by all means, be published.

Since his removal to Mexico, the doctor has made a close study of leprosy; and the paper to which was awarded this splendid prize by the College of Physicians of Philadelphia was the result of his study and observation for the past ten years in Mexico, where he had every facility for making himself familiar with the disease in all its phases.

The paper, which is now, under the terms of the award, the property of the Philadelphia College of Physicians, gives, incidentally, a brief historical sketch of leprosy, and treats of the disease as a specific bacillary affection, uninfluenced by climate, race, occupation, etc., only in so far as these environments tend to enervation on the one hand, or physical vigor on the other; *propagable* by heredity; contagious, infectious and communicable under conditions not yet fully understood; *curable*, *mitigable* and susceptible of *complete eradication* from the list of human ills. The paper details some of the author's personal experience with leprosy patients, and experiments made during the ten years in which he was giving the subject special attention and study. It is illustrated with photographs of some of the subjects experimented on, and such as served to illustrate the different divisions into which the subject was divided; it is also embellished by two beautiful crayon drawings of the *bacillus lepræ*, executed by the doctor's accomplished young wife.

In the preparation of the paper, the author cites the views and opinions of men who are educated in the disease in all its phases; his object apparently being to *elucidate the subject* for the benefit of mankind, rather than to make a display of learning, or to bolster up preconceived opinions of his own; to *arrive at the truth* concerning the nature of a disease known since the beginning of written history, and found among every nation on the globe, but *never understood.*

This paper will become historic, and will constitute a valuable addition to the literature of the subject. It is most opportune, too, as we are threatened on all sides with the disease, and its study is engaging the careful attention of some of our ablest men. Doubtless the paper will appear in due time in the Transactions of the Philadelphia College of Physicians.

Alvarenga was a wealthy Spaniard, who died in Philadelphia, some years ago, leaving a sum of money in the hands of the Philadelphia College of Physicians, the interest of which is to be given each year as a prize for the best paper sent to that body on any medical topic. The committee of award is chosen by the trustees of the fund, and is composed of a representative physician in each state. The prize amounts to about $180.

The JOURNAL extends its cordial congratulations to Dr. Bibb upon this distinguished honor. Dr. Bibb has, from the inception of the enterprise, been a staunch friend and supporter of DANIEL'S TEXAS MEDICAL JOURNAL, and is one of its most valued collaborators. As a writer, he is terse and forcible, rather than florid and prolix; he talks squarely at his subject, and makes every word count.

